# Probiotic-Reduced Inflammaging in Older Adults: A Randomized, Double-Blind, Placebo-Controlled Trial

**DOI:** 10.1007/s12602-024-10310-7

**Published:** 2024-06-19

**Authors:** Irini Lazou-Ahrén, Malin Björklund, Göran Molin, Jie Xu, Gunilla Önning, Sölve Elmståhl, Bengt Jeppsson

**Affiliations:** 1 Probi AB, Lund, Sweden; 2 Department of Process and Life Science Engineering, Lund University, Lund, Sweden; 3 Sapfo Research AB, Bjärred, Sweden; 4 Division of Geriatric Medicine, Department of Clinical Sciences, Lund University, Malmö, Sweden; 5 Department of Surgery, Skåne University Hospital, Lund University, Lund, Sweden

**Keywords:** *Lactiplantibacillus plantarum* HEAL9, *L*. *plantarum*, Low-grade inflammation, Calprotectin, CRP, Older, Inflammaging

## Abstract

The disparity between increased lifespan and healthy aging, marked by prevalent “inflammaging”, highlights the global challenge in care of older persons. This study explored the anti-inflammatory effects of *Lactiplantibacillus plantarum* HEAL9 (LpHEAL9), alone or combined with berries, on older volunteers with chronic low-grade inflammation (LGI). It was a randomized, double-blind, placebo-controlled trial, with a total of 66 volunteers (> 70 years old), randomly assigned, and equally distributed, to placebo, LpHEAL9 or LpHEAL9 + Berries group. After a 2-week run-in period, participants underwent a 4-week dietary intervention. Intake of LpHEAL9 showed a trend towards reduction in serum CRP but without reaching statistical significance. However, LpHEAL9 significantly decreased fecal calprotectin levels compared to placebo. LpHEAL9+Berries did not show any effect on inflammation. Both probiotic groups showed a trend in improving cognitive function albeit not reaching statistical significance. Our findings suggest that the probiotic strain *L*. *plantarum* HEAL9 has a modest impact on LGI in a healthy older population (ClinicalTrials.gov ID: NCT02342496).

**Supplementary Information:**

The online version contains supplementary material available at 10.1007/s12602-024-10310-7.

## Introduction

According to projections from the United Nations [1], the population aged 65 years or older is expected to rise from 0.7 billion in 2019 to 1.5 billion by 2050. Despite advances in healthcare practices, this increase in lifespan has not been paralleled by healthy longevity, presenting significant challenges for healthcare systems globally and adversely affecting the quality of life for individuals. Aging is marked by the accumulation of molecular and cellular alterations that contribute to an elevated vulnerability to chronic diseases prevalent in later life [2]. A key factor underlying various age-related conditions, including atherosclerosis, diabetes, cognitive decline, and Alzheimer’s disease, is the presence of systemic low-grade inflammation (LGI), a status referred to as “inflammaging” [3]. The genesis of LGI is complex and not entirely understood, involving but not limited to genetic predispositions, the biological aging of chromosomal telomeres, oxidative stress, lifestyle factors (e.g., high-fat diet, smoking, sedentary behavior), and alterations in gut microbiota [3–6]. Furthermore, inflammaging is linked to immunosenescence, characterized by the aged immune system’s diminished capacity to generate anti-inflammatory mediators, thereby failing to adequately counteract the release of pro-inflammatory agents, including endotoxins [7, 8].

Emerging evidence indicates an important role of the gut microbiota in contributing to LGI. Altered microbiota has been frequently observed in the metabolic disorders such as obesity and nonalcoholic fatty liver disease (NAFLD), and has been explored as therapeutic target [9]. Aging also brings about significant changes in microbiota composition. For instance, older adults tend to have a decreased proportion of *Bacillota* (also known as Firmicutes) and an increased proportion of *Bacteroidota* (also known as Bacteroidetes) and *Pseudomonadota* (also known as Proteobacteria), along with decreased microbial diversity, compared to younger individuals [10–14]. Such an unfavorable shift could progress to dysbiosis, which in turn leads to increased intestinal permeability to endotoxins (lipopolysaccharides on the surface of Gram-negative bacteria) as well as other bacterial components that can leak into the systemic circulation and eventually precipitate LGI. A worsening of the gut health may also lead to a reduction in nutrient absorption and ultimately resulting in undernourishment of the older individual [15, 16]. Notably, age-associated muscle loss has been linked to increased gut permeability. A recent study reported that a 16-week probiotic intervention repaired intestinal barrier function and improved quality of life measures in older adults suffering muscle decline [17]. Thus, manipulation of the microbiota composition in older population is a promising potential strategy to prevent or reduce age-associated disease and disability, especially when related to low-grade inflammation. Several attempts have been made using probiotics to counteract LGI and have shown promising results. One study found that consuming *Lactobacillus delbrueckii* subsp*. bulgaricus* 8481 over a 6-month period enhanced immune function in older adults (age > 65 years). This was achieved by decelerating the aging process in T cell subpopulations and boosting the immature T cells [18]. Another study showed that intake of *Lactobacillus casei* Shirota strain for 1-month improved innate immune response as measured by natural killer (NK) cell activity in healthy older adults (age 55–74 years) [19]. A dietary intervention incorporating the probiotic VSL#3^®^ containing multiple probiotic strains led to an increase in bifidobacteria, enhanced plasma levels of folate and vitamin B12, and reduced plasma homocysteine levels (marker for oxidative stress) among individuals with low-grade inflammation [20]. Another study testing the effect of multi-strain probiotics coupled with omega-3 showed that 8-week intake of the product increased the anti-inflammatory cytokine IL-10 in older adults with LGI [21]. Moreover, combining probiotics with prebiotics or other functional food ingredients to achieve a synergistic effect presents a highly promising approach to counteract LGI. Ingredients such as antioxidant-rich dark-colored berries have been shown to improve cognitive function in aged rats [22] and counteract oxidative stress in older individuals [23]. One of the bioactive compounds in these berries are tannins which could be degraded by bacterial tannase into smaller phenolic compounds that have anti-inflammatory properties [24].

Considering the strain-specific nature of probiotic functions and their potential in reducing LGI, this study primarily aimed to investigate the anti-inflammatory activity of the probiotic strain *Lactiplantibacillus plantarum* HEAL9 in older individuals exhibiting moderately elevated serum C-reactive protein (CRP) levels, indicative of LGI. While these individuals were otherwise healthy, the primary aim was to understand the direct impact of the probiotic strain *L*. *plantarum* HEAL9 on inflammation markers. *L*. *plantarum* HEAL9 was chosen because it has been previously demonstrated to influence the immune response in combination with another strain (*Lacticaseibacillus paracasei* 8700:2) [25, 26]. Given the significant tannase activity of *L*. *plantarum* HEAL9*,* as a secondary aim, we further examined whether combining this probiotic strain with a mixture of blackberries and blackcurrants enhanced its anti-inflammatory effects, leveraging the bioactive properties of the berries. We employed this dual approach to investigate the probiotic’s standalone benefits and its potential synergistic interactions with polyphenol-rich berries.

## Materials and Methods

### Design of the Study

This was a randomized, double-blind and placebo-controlled study with the objective to evaluate the benefit of *Lactiplantibacillus plantarum* HEAL9 (DSM 15312, LpHEAL9, HEAL9™) alone or in combination with lyophilized blackcurrants and blackberries, compared to a placebo, in older individuals with low grade systemic inflammation. The study was conducted in Sweden (ClinicalTrials.gov ID: NCT02342496) and ethical approval was received by the Ethics committee in Lund/Malmö, Sweden (2014/86). All participants provided signed informed consent before randomization into one of the three study groups. This clinical study was performed in compliance with the Declaration of Helsinki as well as the ICH-GCP guidelines and EU recommendations (CPMP/ICH/135/95).

### Study Participants

The study involved healthy participants of both genders, aged 70 years and above who had systemic low-grade inflammation (LGI). LGI was defined as a CRP level of 2–10 mg/L. Upon enrollment, participants should be able to fill in the study diary daily and understand any oral/written study-related information provided to them. Exclusion criteria included the presence of chronic inflammatory diseases such as IBD and rheumatism, ongoing corticosteroid treatment, and intake of antibiotic treatment in the last 4 weeks prior to inclusion in the study.

### Study Procedures

The study participants were recruited through a database with subjects that had previously taken part in the Epidemiology for Health (EpiHealth) study and had approved of being contacted again for participation in other studies. Following a screening visit to confirm CRP levels, eligible subjects were randomly allocated into one of the three study groups. They entered a 2-week wash-out period during which they refrained from using other probiotic products, and they started to fill in the study diary. After the wash-out period a baseline visit was scheduled for distributing the study product and ensuring the study diary was correctly completed. The participants were instructed not to consume other probiotic products all through the study period. They then returned to the clinic for the final visit 28 days later. The study product was consumed once daily, ideally at breakfast, and the study diary was filled in every day, during the whole intervention period. The parameters recorded in the diary were fecal consistency (Bristol stool form scale), experience of flatulence (none, medium, high) and abdominal pain (none, medium, high). Blood pressure and body weight were measured at each study visit. Plasma and serum samples were collected at baseline and at end of study for analyses of CRP, fibrinogen, ALAT, ASAT, GT, ALP, e-GFR, creatinine, and total protein by Labmedicin Skåne (University and regional laboratories in Region Skåne, Sweden). Additional blood samples were stored at – 80 °C for future analysis of additional blood markers. Serum levels of IFN-γ, IL-10, IL-1β, IL-6, TNF-α, MCP-1, and fractalkine were analyzed using U-PLEX Human assays from MSD (U-PLEX Human, Meso Scale Diagnostics, Rockville, MD, USA) according to the manufacturer’s instructions. Fecal samples were also collected at baseline and at end of study for the analyses of calprotectin (Department of Clinical Chemistry at Skåne University Hospital, Malmö, Sweden) and zonulin using a competitive enzyme-linked immunosorbent assay (ELISA) kit (Immundiagnostik AG, Bensheim, Germany), following the manufacturer’s protocol.

At the start and end of the study, all participants were asked to fill in a quality-of-life questionnaire (SF-36) [27]. The SF-36 is divided into eight multi-items, 0–100 scored scales (physical functioning, role limitations-physical, bodily pain, general health, vitality, social functioning, role limitations-emotional, and mental health). The higher the score, the more positive the health status. Participants’ cognitive function was assessed using the validated Trail Making Test (TMT) [28]. TMT-A determines cognitive processing speed and the subjects had to connect circles labeled with numbers 1–25 in an ascending order. TMT-B measures executive functions and the participants alternated between numbers (1–13) and letters (A-L) in an ascending order (i.e., 1-A-2-B and so forth). A computerized version of TMT was used and administered at baseline and after 28 days intervention (end of study). A computer mouse was used to join the symbols and an erroneous path was indicated by changing its color to red. The total time required to perform the test correctly was measured in seconds. Any adverse events were reported by the participants at the study visits and documented in the case report forms (CRF).

### Investigational Product

The investigational product (IP) was produced in the form of a powder with 10 g per sachet as the daily dose. The active IP consisted of *Lactiplantibacillus plantarum* HEAL9 (LpHEAL9), at 1 × 10^10^ CFU/sachet with or without the addition of a mixture of freeze-dried blackberries and black currants (6 g freeze-dried powder, consisting of 3 g of each berry, derived from 43 g of fresh berries, MOLDA AG, Dahlenburg, Germany). Red beet extract and flavor (Nordarom AB, Norrköping, Sweden) were added to give the study products without berries the same appearance, taste, and texture as the IP with the berries and maltodextrin was used as a filler. The content from each sachet was consumed once daily after mixing with sour milk or pouring over the breakfast flakes. The study participants were randomly allocated to receive one of the three study products (placebo, LpHEAL9, or LpHEAL9 + Berries) using a computer-generated randomization list with blocks of three and the ratio of 1:1:1. Sealed envelopes were prepared for the allocation concealment and were safely stored by the investigators throughout the study. The labelling of the study product and the preparation of the sealed code envelopes were done by personnel not otherwise involved in any study-related activities. Both study participants and study personnel were blinded to the identity of the study product. Compliance was verified based on the daily records of consumption given in the study diary.

### Outcomes

The primary aim of this study was to investigate the impact of *L*. *plantarum* HEAL9 with or without the berries in comparison to placebo, on the changes in CRP levels from baseline to end of study. The secondary objectives included evaluating differences in blood and fecal markers between and within the groups, as measured from baseline to end of study, as well as any changes in blood pressure, body weight, fecal consistency, experience of flatulence, abdominal pain, quality of life, cognition, and adverse events reported.

### Statistical Analyses

The statistical analysis was performed by an independent statistician using StatXact Version 11.1.0, and STATA Version 16. Since no earlier study existed to base the sample size calculation on, it was decided to include about 20 participants per group. The non-parametric Wilcoxon rank sum test was applied for the between groups analysis of continuous variables and when needed Student’s *t* test was used to check the robustness. Fischer’s exact test was used for the categorical endpoints. Analysis of within-group changes was done using Wilcoxon signed rank test for continuous variables and McNemar test for categorical variables. All participants having consumed at least one dose of the investigational products, i.e., the intention to treat (ITT) population, were included in the analysis of safety parameters. The analysis of efficacy parameters was based on the per protocol group (PP) that in this case included all participants from the ITT group except for one participant that had consumed antibiotics during the study. For the serum inflammatory marker analysis, three other participants were excluded due to medical reasons. All presented p values are nominal, i.e., not adjusted for multiplicity, and p values less than 5% are considered statistically significant.

## Results

**Fig. 1:**
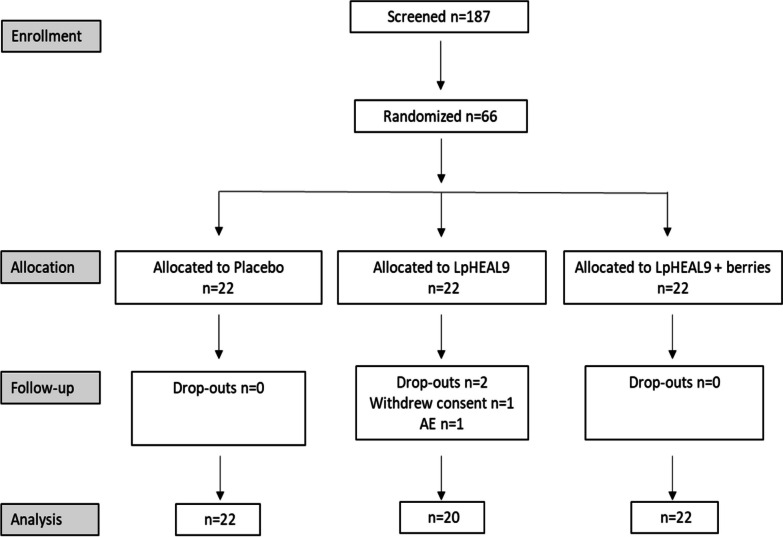
Study flow chart

A total of 394 subjects were contacted to participate in the current study and 187 were screened for eligibility (Fig. 1). In total, 66 participants were included and randomized, 22 participants in each of the three study groups: placebo, LpHEAL9, or LpHEAL9 + Berries. All participants except for two in the LpHEAL9 group completed the study as planned. The reasons for the early terminations were abdominal discomfort in one case and a drop-out for unknown reasons in the other.

There were no differences at baseline between the probiotic groups and the placebo group in terms of gender, age, BMI, and smoking habits or intake of medication (Table 1). The mean age of the subjects was 73.2 years, with a range from 70 to 79 years. Most participants were men (65%) and the mean BMI value of the subjects was 27.8 kg/m^2^ (range 20–43 kg/m^2^). The compliance was good; four participants did not take the study product for 1 day and one participant in the placebo group missed taking the study product for 3 days. Four participants commented that the volume of the study products was large while the rest of the participants did not have any problem with the amount.

**Table 1:** Baseline data, presented as mean ± SD or as number of subjects (%)

	Placebo	LpHEAL9	LpHEAL9 + Berries
Men	16 (73%)	11 (50%)	16 (73%)
Women	6 (27%)	11 (50%)	6 (27%)
Age (years)	73.1 ± 2.1	73.2 ± 1.8	73.2 ± 1.9
BMI (kg/m^2^)	26.4 ± 3.2	28.1 ± 4.4	28.8 ± 4.6
Smokers			
Current	2 (9%)	2 (9%)	2 (9%)
Former	12 (55%)	12 (55%)	12 (55%)
No	8 (36%)	8 (36%)	8 (36%)
Snuff users			
Current	1 (5%)	1 (5%)	1 (5%)
Former	0 (0%)	1 (5%)	0 (0%)
No	21 (95%)	20 (90%)	21 (95%)
Physical activity (≥ 30 min/day)			
Yes	20 (91%)	16 (73%)	18 (82%)
No	2 (9%)	6 (27%)	4 (18%)
Daily intake of alcohol			
Yes	5 (23%)	3 (14%)	4 (18%)
No	17 (77%)	19 (86%)	18 (82%)
Regular intake of products with live bacterial cultures			
Yes	3 (14%)	1 (5%)	7 (32%)
No	19 (86%)	21 (95%)	15 (68%)
Daily intake of medication			
Yes	16 (73%)	16 (73%)	18 (82%)
No	6 (27%)	6 (27%)	4 (18%)
Intake of medication			
Yes	16 (73%)	16 (73%)	18 (82%)
No	6 (27%)	6 (27%)	4 (18%)

### Anti-Inflammatory Activity

CRP levels decreased in the LpHEAL9 group compared to the placebo group but did not reach statistical significance (Table 2, *p* = 0.22). There was a trend of decreasing CRP levels within the LpHEAL9 group (Baseline vs. End of study; median [IQR]: 2.85 [1.68, 5.08] vs. 2.30 [1.13, 3.15] mg/L, *p* = 0.1).

**Table 2:** Change from baseline to end of study in markers of inflammation analyzed in serum (C-reactive protein, fibrinogen, total protein) or feces (calprotectin, zonulin) (median[IQR])

	Placebo			LpHEAL9			LpHEAL9 + Berries			p^a^	p^b^
	Baseline	End of study	Change	Baseline	End of study	Change	Baseline	End of study	Change		
CRP^c^ (mg/L)	2.00 [1.40, 3.30]	2.20 [1.30, 3.80]	0 [− 0.54, 0.50]	2.85 [1.68, 5.08]	2.30 [1.13, 3.15]	− 0.17^f^ [− 1.85, 0.20]	2.30 [1.8, 9.05]	2.90 [2.0, 4.50]	0 [− 0.50, 0.65]	0.22	0.94
Fibrinogen^c^ (g/L)	3.00 [2.70, 3.20]	2.90 [2.60, 3.00]	− 0.05 [− 0.30,0.10]	3.30 [2.80, 3.40]	3.25 [2.68, 3.53]	0.10 [− 0.30, 0.20]	3.00 [2.70, 3.30]	3.10 [2.75, 3.70]	0.20 [− 0.10, 0.30]	0.45	0.06
Total protein^c^ (g/L)	70.00 [68.00, 71.00]	70.00 [67.75, 72.25]	0.50 [− 1.25, 1.25]	68.00 [67.00, 70.00]	69.00 [65.50, 70.75]	0 [− 1.75, 0]	68.00 [65.00, 70.00]	68.00 [64.00, 71.25]	0.50 [− 1.00, 2.00]	0.39	0.52
Zonulin^d^ (ng/ml)	78.64 [62.62, 112.05]	124.15 [65.93, 172.01]	− 5.07 [− 18.91, 67.98]	96.62 [72.95, 135.20]	100.91 [79.88, 129.00]	− 1.53 [− 22.22,14.56]	84.53 [46.97, 114.07]	77.00 [51.68, 140.33]	10.28^f^ [− 9.41, 30.56]	0.75	0.49
Calprotectin^d^ (mg/kg)	24.50 [24.00, 47.75]	32.00 [24.00, 61.75]	1.00 [− 0.75, 11.75]	36.00 [24.00, 101.00]	35.00 [24.00, 66]	− 1.00 [− 9.00, 0]	31.50 [24.00, 103.75]	34.50 [24.00, 105.75]	0 [− 9.75, 13.75]	0.03	0.35

^a^*p* value for the comparison of the change in LpHEAL9 group with placebo based on Wilcoxon rank sum test

^b^*p* value for the comparison of the change in LpHEAL9 + Berries with the placebo based on Wilcoxon rank sum test

^c^Analysis excluding one subject from each group due to the common cold or antibiotics intake

^d^Analysis excluding two subjects with high values due to medical reasons

^f^A trend (*p* = 0.1) compared to baseline within-group based on Wilcoxon signed rank test

**Fig. 2:**
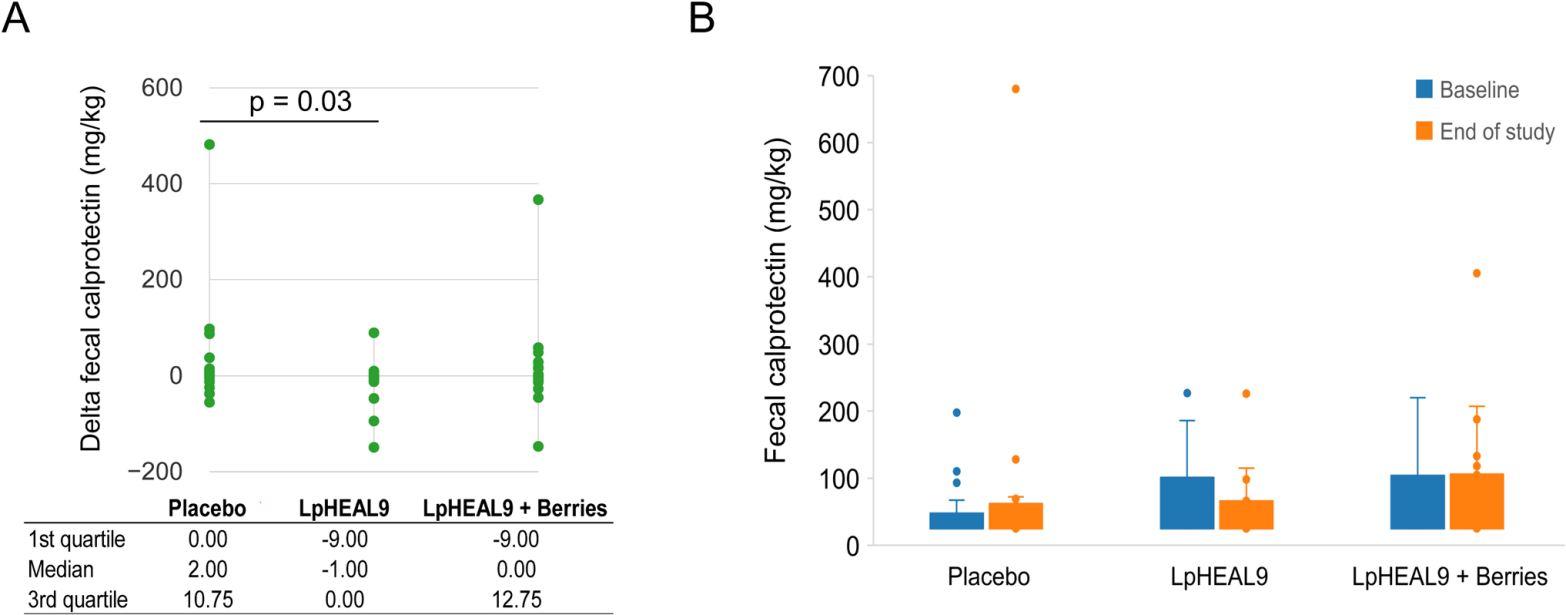
Fecal calprotectin levels in the elderly participants. Changes in the fecal calprotectin levels from baseline to the end of study showed a significant reduction in LpHEAL9 group compared to placebo (**A**). Measured calprotectin levels at baseline and end of study in each group (**B**). No significant difference was detected within-group comparisons

Fecal calprotectin levels decreased significantly after the consumption of LpHEAL9 compared to placebo (Table 2, Fig. 2). Moreover, there was a significant difference (*p* = 0.01) in the number of participants with increased calprotectin values at end of study compared to baseline in the placebo group (11/22) *versus* the LpHEAL9 group (2/17). No significant differences were found within the groups in calprotectin levels for baseline *versus* end of study.

No significant differences were identified between the probiotic groups and the placebo group regarding any changes in fibrinogen and total protein measured in serum, or zonulin measured in feces (Table 2). The possible anti-inflammatory activity of the probiotic bacteria was further assessed in serum with the analyses of IFN-g, IL-10, IL-1b, IL-6, TNF-a, MCP-1, and fractalkine. No significant differences were identified in the probiotic groups compared to the placebo group for any of these markers (Table 3). However, in terms of change from baseline to end of study in the levels of TNF-a, the reduction in the LpHEAL9 group (median [IQR], − 0.10 [− 0.37, 0.52]) pg/ml tended (p = 0.07) to differ from that seen in the placebo group (median [IQR], 0.09 [− 0.21, 0.60] pg/ml).

**Table 3:** Change from baseline to end of study (Δ) in different biomarkers (median [min, max], pg/ml)

	Placebo (Δ)	LpHEAL9 (Δ)	LpHEAL9 + Berries (Δ)	p^a^	p^b^
IFN-γ	− 0.03 [− 6.95, 26.33]	0.26 [− 4.36, 4.51]	0.40 [− 29.32, 8.34]	0.75	0.65
IL-10	− 0.01 [− 0.06, 1.44]	0.01 [− 0.16, 0.08]	− 0.01 [− 2.92, 0.46]	0.90	0.82
IL-1β	0 [− 0.09, 0.06]	− 0.01 [− 1.03, 6.49]	− 0.01 [− 0.38, 0.26]	0.93	0.68
IL-6	0.04 [− 0.53, 1.05]	0.11 [− 0.63, 7.23]	0 [− 4.45, − 0.24]	0.45	0.45
TNF-α	0.09 [− 0.21, 0.60]	− 0.10 [− 0.37, 0.52]	0.06 [− 1.10, 8.56]	0.07	0.85
Fractalkine	267.06 [− 1681.00, 3750.00]	151.36 [− 656.91, 734.00]	− 155.89 [− 1976.00, 1967.00]	0.70	0.12
MCP-1	− 5.00 [− 72.39, 53.75]	11.47 [− 125.36, 74.00]	6.00 [− 188.20, 89.00]	0.35	0.41

^a^*p* value for the comparison of the LpHEAL9 group with placebo based on Wilcoxon rank sum test

^b^*p* value for the comparison of the LpHEAL9 + Berries with the placebo based on Wilcoxon rank sum test

### Gastrointestinal Symptoms, Quality of Life, and Safety Markers

There were no differences between the probiotic groups and the placebo group in number of bowel movements at start or at any other measured timepoints in the study (data not shown). For the LpHEAL9 group the number of bowel movements increased significantly at week 2 of the intervention compared to baseline but this difference was not sustained in the following weeks (Fig. S1A). In the same group, a significant decrease in the mean weekly number of normal stools (i.e., the sum of Bristol stool types 3, 4, and 5) compared to baseline was observed at week 1, 2, and 3. No significant changes in stool consistency compared to baseline were observed in the other study groups (Fig. S1B).

The SF-36 questionnaire was used to evaluate quality of life, where a higher value indicates a better health status (Table S1). The social functioning decreased in all three groups, with a significant decrease within the LpHEAL9 (*p* < 0.05) and a trend of reduction in the LpHEAL9 + Berries group (*p* < 0.1). However, there were no statistically significant differences found between the groups. Bodily pain was significantly improved (*p* < 0.05) within the LpHEAL9 + Berries group, and the improvement from baseline to end of study was significantly larger compared to the placebo group.

There were no alterations in the level of flatulence, abdominal pain, liver (ASAT, ALAT, GT, ALP) or kidney function (eGFR) and body weight, in any of the groups during the study period (Table S2). In terms of blood pressure, no change over time was detected in the two probiotic groups, whereas there was a significant reduction seen for SBP in the placebo group. This resulted in a significant difference in the change of SBP between LpHEAL9 group and placebo group (*p* = 0.01) (Table S2).

### Cognitive Function

The cognitive function was evaluated at baseline and end of the study (week 4) with trail making test A (TMT-A) and B (TMT-B) (Table S3). The time for making the TMT-A correctly at baseline among all participants ranged from 28 to 208 s and for TMT-B the range was from 35 to 237 s with mean values of 47.49 and 74.71 s, respectively. No significant differences were detected in the between-group comparisons. However, a trend toward improved cognitive function over time was observed within the LpHEAL9 group, as evidenced by a decrease in the time required to perform TMT-A (*p* = 0.092). A similar trend was observed for TMT-B in the LpHEAL9 + Berries group (*p* = 0.067).

## Discussion

This study aimed to assess the anti-inflammatory effects of the probiotic strain *L*. *plantarum* HEAL9 (LpHEAL9), either standalone or in combination with lyophilized berries, in older individuals with low-grade systemic inflammation (LGI). Chronic LGI is a recognized characteristic of inflammaging, yet standard biomarkers to definitively indicate the presence of this LGI state are lacking [29]. Consequently, researchers often rely on a variety of biomarkers, with standard CRP or high-sensitivity CRP (hs-CRP) as a common endpoint to identify LGI. Our findings indicate a trend towards reduced CRP levels in the LpHEAL9 group compared to the placebo group, hinting at a potential anti-inflammatory role for this probiotic strain. Nevertheless, the absence of statistical significance calls for larger future studies to determine the efficacy of LpHEAL9 in lowering CRP levels. To note, at baseline—2 weeks post-inclusion and prior to the intake of study products—approximately 36% of participants (32% in both the LpHEAL9 and LpHEAL9 + Berries groups, and 45% in the placebo group) exhibited CRP levels below 2 mg/L, indicating a lack of low-grade inflammation at the intervention’s outset, which poses a limitation to our study. Previous studies have shown a more pronounced reduction in hs-CRP levels following probiotic supplementation when participants were stratified by an hs-CRP ≥ 3 mg/L cutoff, indicative of LGI [20]. This demonstrates a critical influence of baseline inflammatory status on research outcomes and could partially explain why we did not see an effect on CRP. On the other hand, a modest sample size of the present study restricted our ability to perform detailed subgroup analyses, underlining the inherent difficulties of relying on CRP levels for defining low-grade systemic inflammation. Additionally, the within person variability of CRP levels in a short timeframe, also observed with hs-CRP [18, 23], further complicates the assessment. This variability highlights the necessity for more precisely defined biomarkers to accurately define the low-grade inflammation state.

Intake of LpHEAL9 for 4 weeks resulted in significantly reduced levels of fecal calprotectin in comparison with the placebo group. Further analysis showed that significantly more participants in the placebo group, compared to the LpHEAL9 group, had an increase in the calprotectin level. In the context of chronic inflammation, elevated fecal calprotectin level can reflect both neutrophil migration through an inflamed gut wall and upregulated expression of the calprotectin by intestinal epithelial cells in response to inflammatory stimuli [30]. Notably, fecal calprotectin was superior in distinguishing between non-inflammatory and inflammatory bowel disease compared to CRP [31–33]. A 1-month intervention study reported that 15 days of consuming the Mediterranean diet did not affect fecal calprotectin levels in older obese women, but combining the Mediterranean diet with multi-strain probiotics during the following 15 days led to a significant reduction in fecal calprotectin levels [34]. Another study involving middle aged individuals with functional diarrhea and elevated fecal calprotectin demonstrated that the administration of a different *L*. *plantarum* strain (CJLP243) over an 8-week period significantly reduced calprotectin levels from baseline [35]. Concurrently, a greater proportion of participants in the probiotic group experienced substantial relief from functional diarrhea (measured as more than 30% reduction in the frequency of loose stools) compared to those in the placebo group [35]. On the other hand, administration of probiotic strain *Bacillus coagulans* GBI-30, 6086 had no effect on fecal calprotectin level in older participants but increased anti-inflammatory cytokine IL-10 [36]. These inconsistencies in reducing calprotectin level via probiotics intervention could be due to the strain dependent effect or, as already mentioned, to variation in baseline inflammatory status in the studied population. While universal reference values for inflammation are not established, fecal calprotectin level below 40 mg/kg suggests a ≤ 1% chance of inflammatory bowel disease [27]. For the calprotectin measurement technique utilized in this study, a reference value of < 50 mg/kg was adopted, with 63% of participants exhibiting values beneath this threshold at baseline.

We hypothesized that by combining tannase-producing LpHEAL9 with tannin-rich blackberries and blackcurrants would result in smaller phenolic compounds with anti-oxidative and anti-inflammatory capacity [24, 37], which in turn could deliver extra benefit. However, in participants consuming the LpHEAL9 + Berries there was no obvious benefit in CRP levels, calprotectin or other inflammatory markers compared to the participants that consumed LpHEAL9 alone. The amount of freeze-dried berries in the daily dose of the IP corresponded to 43 g fresh berries and this may be a too low dose to observe an effect on inflammation. A study on obese subjects with increased LDL cholesterol levels, showed that consuming high dose strawberry powders (32 g/day, equivalent to ~ 375 g of fresh strawberries) but not low dose (equivalent to one serving daily of ~ 150 g fresh strawberries), reduced the level of the an inflammatory marker serum plasminogen activator inhibitor-1 (PAI-1) [38].

Proinflammatory cytokine TNF-α is another frequently measured marker in intervention studies targeting inflammation. Although there is no consistent evidence of probiotic effect on TNF-α [18, 20, 21, 36, 39, 40], a meta-analysis on probiotics effect on inflammatory markers found that probiotic consumption could significantly reduce TNF-α, and also CRP and IL-6 [41]. In the current study, a trend was observed toward a reduction in the proinflammatory TNF-α in LpHEAL9 group compared to placebo.

In the current study, we have observed tendencies towards improved performance in trail making tests used to examine visual attention and executive functioning within both probiotic groups. A recent finding also showed that the same probiotic strain as used in the current study, LpHEAL9, enhanced cognitive functions, mood, and sleep in moderately stressed but otherwise healthy individuals aged 21 to 52 years [42]. The small sample size and low statistical power might contribute to lack of significant differences between groups. There is a growing interest in exploring the gut-brain axis as a means to understand how altering the gut microbiota can impact neurological function [43, 44]. This interest is driven by the potential of using gut microbiota modulation as a non-pharmacological strategy to combat cognitive decline that often accompanies aging. It has been reported that 12-weeks intake of probiotics containing bifidobacteria strains led to an improvement in mental flexibility and mood in healthy older persons [45].

Regarding product safety, our findings contribute to the growing body of evidence supporting the safety of probiotic supplementation in older populations. The overall good tolerance reported by most participants, alongside the absence of significant changes in gastrointestinal symptoms, liver and kidney function, and on body weight across all study groups, demonstrate the safety profile of LpHEAL9, both alone and in combination with lyophilized berries. This is particularly relevant given the heightened vulnerability of the older persons to adverse health effects from supplements and the critical importance of ensuring safety in interventions targeting this population.

## Conclusion

In summary, a 4-week intake of LpHEAL9 significantly decreased levels of fecal calprotectin in this group of otherwise healthy older subjects, indicating an anti-inflammatory effect. Additionally, the LpHEAL9 probiotic strain has shown promising trends toward reducing CRP and TNF-α levels and improving cognitive function, underscoring its potential to support healthy aging by modulating inflammation. The inclusion of tannin-rich blackberries and blackcurrants with the LpHEAL9 strain did not provide the expected synergistic benefits. This could be due to the relatively low dose of berries used in the study, potentially limiting their ability to enhance the probiotic’s effects. Further research is necessary to fully explore the impact of the LpHEAL9 strain on low-grade inflammation and its benefits for healthy aging, including investigations into optimal dosages of berry supplementation for achieving synergistic effects.

## Supplementary Information

Below is the link to the electronic supplementary material.

Supplementary file1 (TIF 70284 KB)

Supplementary file2 (DOCX 28 KB)

## Data Availability

Supporting data for the findings of this study are available upon request from the corresponding author.
