# Supplementary material for: Probiotic-Reduced Inflammaging in Older Adults: A Randomized, Double-Blind, Placebo-Controlled Trial
Source: Probiotics Antimicrob Proteins. 2024 Jun 19;17(5):3429–39. doi: 10.1007/s12602-024-10310-7 (PMC12532685; doi:10.1007/s12602-024-10310-7)
Supplement: Supplementary file 2 — Supplementary file2 (DOCX 28 KB) [file 12602_2024_10310_MOESM2_ESM.docx]

Table S1. SF-36 Item Health Survey 1.0

|  | Placebo | | |  | LpHEAL9 | | |  | LpHEAL9 + Berries | | |
| --- | --- | --- | --- | --- | --- | --- | --- | --- | --- | --- | --- |
|  | Start | End | Change |  | Start | End | Change |  | Start | End | Change |
| Cluster 1:  Physical functioning | 86.6 ± 13.1 | 84.4 ± 15.8 | -2.4 ± 6.8 |  | 67.7 ± 26.4^#^ | 70.5 ± 24.9^#^ | 1.5 ± 9.3 |  | 80.7 ± 21.8 | 81.2 ± 19.7 | -0.51 ± 7.5 |
| Cluster 2:  Role limitations due to physical health | 82.6 ± 33.1 | 86.3 ± 31.9 | 5.0 ± 37.7 |  | 75.0 ± 40.8 | 73.8 ± 35.8 | -4.8 ± 20.3 |  | 84.5 ± 30.1 | 85.2 ± 32.4 | 0.0 ± 34.5 |
| Cluster 3:  Role limitations due to emotional problems | 90.2 ± 22.2 | 91.7 ± 26.2 | 2.5 ± 15.6 |  | 82.6 ± 35.8 | 77.8 ± 35.5 | -6.3 ± 25.0 |  | 96.8 ± 10.0 | 89.4 ± 23.9 | -7.9 ± 27.7 |
| Cluster 4:  Energy/fatigue | 79.3 ± 15.3 | 78.5 ± 18.2 | -0.79 ± 12.4 |  | 66.6 ± 26.1 | 70.0 ± 24.1 | 1.2 ± 9.3 |  | 76.4 ± 21.6 | 76.9 ± 21.7 | 0.53 ± 20.0 |
| Cluster 5:  Emotional well-being | 90.5 ± 11.8 | 89.4 ± 15.0 | -1.0 ± 7.9 |  | 82.4 ± 19.7 | 85.0 ± 16.8 | 1.5 ± 6.3† |  | 89.2 ± 11.1 | 89.5 ± 13.2 | 0.27 ± 9.1 |
| Cluster 6:  Social functioning | 94.9 ± 10.7 | 86.3 ± 16.7 | -8.1 ± 14.4 |  | 89.8 ± 17.1 | 82.0 ± 20.0 | -8.4 ± 10.7* |  | 92.6 ± 16.7 | 83.3 ± 19.3 | -9.3 ± 20.7^§^ |
| Cluster 7:  Pain | 79.9 ± 21.7  90 (23, 100) | 79.4 ± 24.0  90 (23, 100) | -0.75 ± 25.0  0 (-35, 78) |  | 57.0 ± 26.7^#^  58 (0, 100) | 62.1 ± 25.4^#^  58 (23, 100) | 3.1 ± 21.0 |  | 76.0 ± 23.6 | 83.6 ± 20.0 | 7.6 ± 11.7*^#^  0 (0, 45) |
| Cluster 8:  General health | 72.1 ± 16.7 | 75.5 ± 18.7 | 3.7 ± 12.2  0 (-20, 35) |  | 67.5 ± 20.5 | 71.4 ± 19.6 | 2.9 ± 11.0 |  | 75.4 ± 21.8 | 73.1 ± 17.8 | -2.2 ± 10.7^#^  -5 (-20, 32) |

*Significant change over time within the group (p<0.05), ^§^trend for change over time within the group (p<0.1), ^#^significant difference compared to placebo (p<0.05), †trend for difference compared to placebo (p<0.1), values are presented as means ± SD but median (min, max) is also shown for cases with a significant difference compared to placebo based on Wilcoxon rank sum test.

Table S2: Change in safety markers, blood pressure and weight during the study. Values are presented as mean ± SD

|  | Placebo | | |  | LpHEAL9 | | |  | LpHEAL9 + Berries | | |  |  |
| --- | --- | --- | --- | --- | --- | --- | --- | --- | --- | --- | --- | --- | --- |
|  | Baseline | End | Change |  | Baseline | End | Change |  | Baseline | End | Change | p^1^ | p^2^ |
| SBP (mmHg) | 146.1 ± 17.0 | 140.3 ± 18.9 | -5.8 ± 11.0* |  | 134.0 ± 11.6 | 135.5 ± 16.2 | 1.7 ± 11.8 |  | 140.3 ± 16.9 | 140.8 ± 16.8 | -0.55 ± 11.14 | 0.01 | 0.06 |
| DBP (mmHg) | 79.9 ± 9.2 | 77.6 ± 9.0 | -2.3 ± 6.0 |  | 75.5 ± 10.8 | 76.0 ± 9.3 | 0.9 ± 8.0 |  | 76.9 ± 8.2 | 77.8 ± 7.0 | 1.0 ± 6.4 | 0.16 | 0.10 |
| ALAT (mkat/l) | 0.42 ± 0.15 | 0.43 ± 0.16 | 0.01 ± 0.10 |  | 0.44 ± 0.35 | 0.35 ± 0.11 | -0.01 ± 0.07 |  | 0.38 ± 0.12 | 0.33 ± 0.08 | -0.05 ± 0.09 | 0.46 | 1 |
| ASAT (mkat/l) | 0.44 ± 0.1 | 0.45 ± 0.10 | 0.01 ± 0.09 |  | 0.42 ± 0.16 | 0.37 ± 0.09 | -0.01 ± 0.07 |  | 0.41 ± 0.07 | 0.40 ± 0.06 | -0.01 ± 0.07 | 0.78 | 0.58 |
| GT (mkat/l) | 0.60 ± 0.39 | 0.61 ± 0.35 | 0.01 ± 0.12 |  | 0.59 ± 0.39 | 0.43 ± 0.21 | -0.08 ± 0.22 |  | 0.71 ± 0.62 | 0.75 ± 0.66 | 0.04 ± 0.18 | 0.53 | 0.72 |
| ALP (mkat/l) | 1.08 ± 0.26 | 1.08 ± 0.30 | 0.0 ± 0.24 |  | 1.27 ± 0.45 | 1.28 ± 0.41 | -0.07 ± 0.25 |  | 1.14 ± 0.26 | 1.15 ± 0.29 | 0.01 ± 0.10 | 0.44 | 0.92 |
| eGFR(ml/min/1.73 m^2^) | 82.0 ± 66.45 | 81 ± 66.9 | 17.0 ± 0.45 |  | 90.0 ± 69.1 | 90.0 ± 69.9 | 8.0 ± 0.0 |  | 65.1 ± 7.8 | 64.1 ± 9.1 | -0.9 ± 5.1 | 0.80 | 0.53 |
| Body weight (kg) | 79.6 ± 13.4 | 79.8 ± 13.5 | 0.15 ± 1.33 |  | 80.6 ± 13.5 | 81.39 ± 13.9 | 0.06 ± 0.79 |  | 84.7 ± 17.5 | 84.5 ± 17.3 | -0.25 ± 1.17 | 0.80 | 0.27 |

^1^ p-value for the comparison of the LpHEAL9 group with placebo based on Wilcoxon rank sum test, ^2^ p-value for the comparison of the LpHEAL9 + Berries with the placebo, * significant change over time within the groups (p<0.05).

Table S3: Change in the performance on trail making test A (TMT-A) and B (TMT-B). Values are presented as mean ± SD^1^

|  | TMT-A (sec) | | | |  | TMT-B (sec) | | | |
| --- | --- | --- | --- | --- | --- | --- | --- | --- | --- |
|  | Baseline | End | Change | p^2^ |  | Baseline | End | Change | p^2^ |
| Placebo (n=22) | 45.75 ± 10.21 | 47.75 ± 28.05 | 2.01 ± 27.48 | 0.735 |  | 84.36 ± 44.08 | 83.10 ± 66.66 | -1.26 ± 36.81 | 0.874 |
| LpHEAL9 (n=18) | 46.55 ± 14.97 | 41.61 ± 10.00 | -4.94 ± 11.72 | 0.092 |  | 66.41 ± 25.48 | 65.27 ± 26.09 | -1.14 ± 23.83 | 0.841 |
| LpHEAL9 + Berries (n=22) | 50.00 ± 37.12 | 42.62 ± 13.90 | -7.38 ± 26.88 | 0.212 |  | 71.86 ± 31.85 | 64.14 ± 28.01 | -7.73 ± 18.75 | 0.067 |

^1^ There was no difference between the groups at baseline or in change from baseline (t-test).

^2^ p-value for the comparison of change from baseline within a group (paired t-test).
